# Personalized Pseudophakic Model for Refractive Assessment

**DOI:** 10.1371/journal.pone.0046780

**Published:** 2012-10-03

**Authors:** Filomena J. Ribeiro, António Castanheira-Dinis, João M. Dias

**Affiliations:** 1 GoLP/Instituto de Plasmas e Fusão Nuclear-Laboratório Associado, Instituto Superior Técnico, Lisbon, Portugal; 2 Visual Sciences Research Centre, University of Lisbon, Lisbon, Portugal; 3 Hospital da Luz, Lisbon, Portugal; Univeristy of Melbourne, Australia

## Abstract

**Purpose:**

To test a pseudophakic eye model that allows for intraocular lens power (IOL) calculation, both in normal eyes and in extreme conditions, such as post-LASIK.

**Methods:**

Participants: The model’s efficacy was tested in 54 participants (104 eyes) who underwent LASIK and were assessed before and after surgery, thus allowing to test the same method in the same eye after only changing corneal topography.

**Modelling:**

The Liou-Brennan eye model was used as a starting point, and biometric values were replaced by individual measurements. Detailed corneal surface data were obtained from topography (Orbscan®) and a grid of elevation values was used to define corneal surfaces in an optical ray-tracing software (Zemax®). To determine IOL power, optimization criteria based on values of the modulation transfer function (MTF) weighted according to contrast sensitivity function (CSF), were applied.

**Results:**

Pre-operative refractive assessment calculated by our eye model correlated very strongly with SRK/T (r = 0.959, p<0.001) with no difference of average values (16.9±2.9 vs 17.1±2.9 D, p>0.05). Comparison of post-operative refractive assessment obtained using our eye model with the average of currently used formulas showed a strong correlation (r = 0.778, p<0.001), with no difference of average values (21.5±1.7 vs 21.8±1.6 D, p>0.05).

**Conclusions:**

Results suggest that personalized pseudophakic eye models and ray-tracing allow for the use of the same methodology, regardless of previous LASIK, independent of population averages and commonly used regression correction factors, which represents a clinical advantage.

## Introduction

Modelling the optics of an individual patient's eye, and predicting the resulting visual performance are major goals for visual optics and clinical researchers. Although generic eye models are of great use, they do not reflect individual anatomical characteristics, and are thus limited. Therefore, the development of personalized models, using individual biometry data and encompassing individual aberrations [1], address a currently unmet need. The improvement of IOL power calculation after corneal refractive surgery is an issue that is becoming increasingly important, due to the recognition that currently used formulas do not provide an adequate prediction, often resulting in falsely low IOL power [2]. Although several approaches have been developed to minimize IOL power calculation error caused by corneal power misevaluation [3], some methods require previous clinical history data, which is frequently not available, while others use correction factors specific for a certain measurement technique or equipment [4].

Wavefront technology and ray tracing are very promising technologies that have been used to improve IOL power calculation error [5]–[7], since they describe better the optics of the pseudophakic eye. Ray-tracing allows for exact calculations, retaining only the errors inherent to biometric measurements, being a better competitor compared with paraxial optical methods, as long as the studied eye is properly modulated. Despite those advantages, and in order to achieve the proper eye modelling for ray-tracing, there are still some needs to be fulfilled: 1) a better description of the corneal surface, especially in cases of non-spherical post-LASIK corneas [8] and 2) a better definition of optimization metrics to determine the best image plane [1].

In this paper, we report the design of a personalized pseudophakic model which overcomes those two needs by using a full 3-D definition of the cornea based on detailed corneal elevation data, obtained from topography, and an optimization metric based on the MTF and CSF. The model was tested in 54 participants (104 eyes) who were assessed before and after LASIK, thus allowing to test the same method in the same eye after only changing corneal topography. In order to evaluate the efficacy of the model, results were compared to currently used methods of IOL power calculation.

## Methods

### Population Sample for Model Testing

54 participants (104 eyes), with average age of 33.8±8.0 years, with pre-LASIK refraction of –3.07±1.95 D, corneal anterior radii of 7.74±0.26 mm, ACD of 3.04±0.31 mm (distance from corneal endothelium to lens), lens thickness of 3.87±0.36 mm and vitreous chamber depth of 16.96±1.05 mm, scheduled to undergo LASIK refractive surgery, were assessed before and 1 month after LASIK. Topography data was obtained with an Orbscan II® (Bausch and Lomb Inc., Rochester, NY, USA) and contact biometry data without immersion with Ocuscan® (Ultrasound biometry Alcon RxP). Given Orbscan II® is based on slit scan beam imaging and uses mathematical calculations to recreate the posterior cornea, this strategy can cause false positive readings of posterior corneal elevation [9], and its accuracy for assessing posterior corneal measurements, especially in post-LASIK situations, has been questioned [10]–[12]. More experimental data on corneal optical and biometric properties, and more accurate models of corneal biomechanics should be studied to provide better information of corneal shape in post-LASIK cases. However, our method is prepared to readily incorporate data from any currently existent more precise technology [12] or future techniques for measuring corneal parameters. Inclusion and exclusion criteria were the recommended for LASIK surgery. The study protocol was approved by Hospital da Luz Institutional Review Board. All participants provided written informed consent.

### Modeling

#### General model definitions

The Liou-Brennan eye model [13] was used as a starting point, since it is the most anatomically accurate considering the biometric and optical data of the physiological eye. It is a finite, non-paraxial model, being the most realistic concerning the average of aberrations of the physiologic eye [14]; [15]. As defined by this model, pupil decentration was set at 0.5 mm from the optical axis with a 5° angle between the visual and optical axis. We have incorporated these parameters in our model due to the growing body of evidence that emphasizes the consideration of angle kappa and pupil decentration in refractive surgery [16]; [17], and our model is prepared to incorporate all these parameters in a personalized manner, as data becomes available in the clinical practice. The Stiles-Crawford effect was incorporated using the formula I = 10^−(α/2)r^2^^, where I is the beam intensity, α = 0.05 and r the radial distance to the pupil centre, in mm [18]. Receptor photopic spectral sensitivity was simulated using 510, 555 and 610 nm wavelengths, with relative weights of 1, 2 and 1, respectively [13]. After inserting into the model the custom glasses representing the different optical elements, and in order to calculate refractive indexes according to wavelength, thus taking chromatic dispersion into account, the chromatic dispersion curve, n(medium at λ[µm]) = n (medium at λ = 0.555 µm)+0.0512–0.1455λ+0.0961λ^2^, defined by Liou-Brennan [13], was fitted using the methods proposed by the Zemax® catalogue (Zemax® is an optical design program from Radiant Zemax®, LLC, Redmond, Washington, USA). Although the Liou-Brennan model does not define a pupil diameter, this parameter has been reported as the forth major source of error in refractive assessment, when considering spherical aberration [19]. Therefore, pupil diameter was set at 4.0 mm, which is representative of the conditions in which refraction is usually measured in clinical practice [20], and defined as the stop surface, which controlled the size and shape of the incoming optical rays. Moreover, effective corneal power increases with pupil size as a result of the spherical aberration [21], with variations that can be significant depending on existing aberrations [5]. Although the Stiles-Crawford effect tends to correct for the spherical aberration of the ocular system [8], only aspherical IOLs in which the asphericity is fitted to the eye in such a way that the resulting spherical aberration is zero do not show a pupil width–dependent focus shift. Our model is prepared to incorporate pupil diameter in a personalized manner. Off-axis monochromatic aberrations were taken into account by considering a spherical retina with a 12 mm curvature radius. All these parameter definitions were retained on our model since it is not common practice to measure them.

#### Personalized model definitions

**Figure 1:**
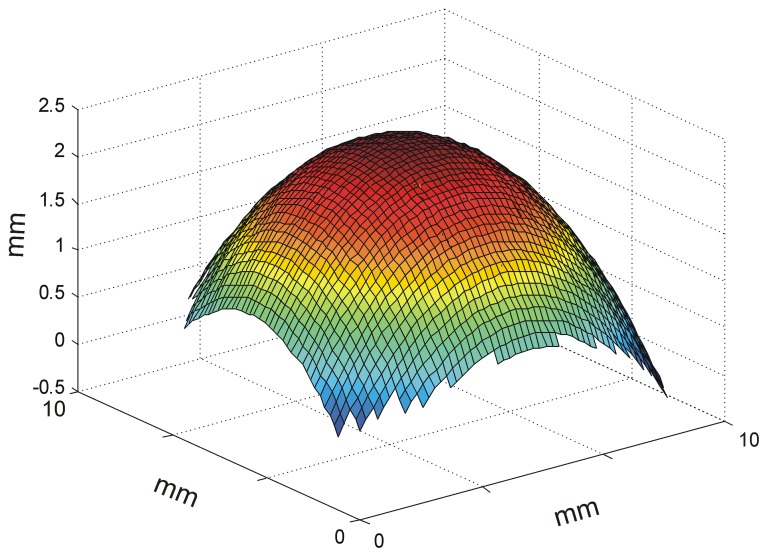
Interpolated corneal elevation data. Tridimensional corneal representation. Corneal elevation data generated from topography was re-formatted and imported to Zemax®. Afterwards, a full definition of the surface shape was obtained through a bicubic spline interpolation of the imported data, thus allowing for ray-tracing. xx and yy axis represent value distribution of the grid over a corneal surface of 10 mm, zz axis represents elevation values.

In order to define individual corneal surfaces, a full 59×59 grid of elevation values from corneal anterior and posterior surface data were obtained from topography with an Orbscan II® and using a 10 mm diameter. Elevation data allow a full spatial morphological description of the corneal surface [22], since it reflects the real tridimensional corneal geometry (Figure 1). Corneal elevation data was converted into a format compatible with Zemax® using Matlab® (matrix laboratory, developed by MathWorks), after developing a software that would allow the correct data format to be generated from OrbScan II® data. The tridimensional surface shape was then determined by a bicubic spline interpolation of the sag values. To test the efficacy of the corneal representation of our model, we have done a best sphere fit to corneal anterior surface data from our model and compared the values obtained with values measured by keratometry, since this is the method used in clinical practice for pre-LASIK cases.

#### IOL definition

Since one of the goals of our model is IOL calculation, we have used pre-operative data to estimate post-operative anterior chamber depth (ACD_post_). In order to define the IOL lens position, ACD_post_, taken as the distance from the corneal endothelium to the anterior IOL surface, was calculated using the measured pre-operative ACD and lens thickness (LT) and considering IOL position at the lens equator defined by the Liou-Brennan model. Hence, the used formula was: ACD_post_ = ACD+0,395 LT. IOL was defined by its geometry - anterior and posterior curvature radius, thickness and refraction index, according to the catalogue of the AR40_e_ (AMO) ® IOL. It should be noted that ACD_post_ will always be an estimation, since it cannot be physically measured before IOL placement. Since our aim was that our eye model would be as independent as possible from regressive factors derived from population studies, ACD_post_ estimation was based solely on biometric values and the definition of equator lens.

#### Optical analysis

The optical software Zemax® was used to construct a pseudophakic eye model. Once the virtual eye is defined, this software uses wavefront technology and exact ray-tracing to modulate light propagation through the optic system to the surface defined as image – the retina. The resulting amplitude distribution and phase of a ray beam allow the analysis of different optic phenomena.

#### IOL selection procedure

This model takes into consideration the optical aberrations that limit the quality of the human eye retinal image, and an optimization procedure has to be adopted in order to choose the best corrective solution. In fact, the optimization procedure is the key process for the calculations of optical components in the virtual eye through the minimization of a predefined merit function. Although it is unknown which criteria the human eye actually uses for focusing, and as such the ideal optimization method is yet to be determined, wavefront root-mean-square (RMS) minimization has been the most commonly used optimization criterion of best focus plane in ray-tracing. However, previous studies have shown that it does not correspond to subjective refraction, always retaining a significant amount of residual Zernike defocus [23], and leading to a myopic eye [24]. Therefore, we have chosen a merit function defined in order to minimize the difference of the MTF values in respect to the diffraction limit values, attributing different weights to each frequency (up to 100 cycles/mm, which corresponds to Snellen’s 10/10 visual acuity), according to the CSF. This metric was chosen because previous studies have shown that one of the best metrics to estimate defocus is the VSMTF [25]. Moreover, this metric also takes into account the facts that different frequencies respond differently to defocus [26] and neuronal sensitivity varies with frequency [27], and in accordance to channel theory, which establishes that the visual pathway decomposes light in frequencies [28]. The chosen image metrics incorporates all these variables and takes into account not only the optics of the human eye but also neuronal factors.

**Figure 2:**
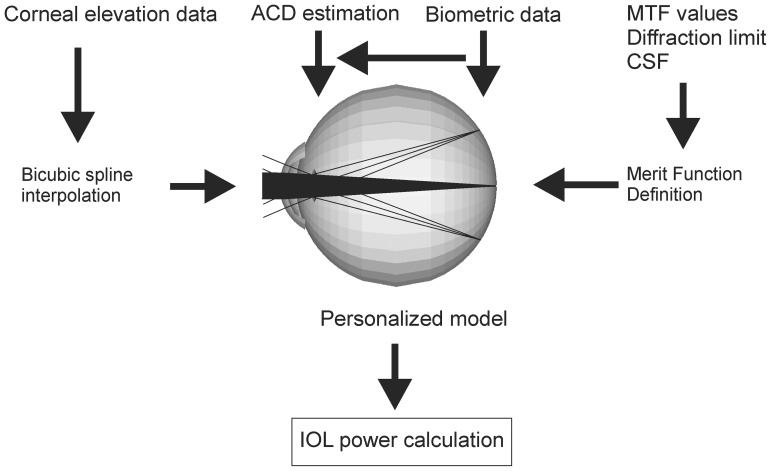
Overview of the developed Personalized Pseudophakic Model. With the schematically represented algorithm, an individual pseudophakic model was obtained for each of the 104 assessed eyes, both before and after refractive surgery. Individual ray-tracing was then performed to allow IOL power calculation.

A schematic representation of the various inputs of the personalized model introduced in the optical analysis software described above is shown in figure 2.

### Statistical Analysis

Correlations were assessed using the Pearson correlation coefficient. Linear regressions of the form y = Bx+A were performed and standard errors σ of all parameters were calculated. Means were compared using t-tests. Tests were considered significant at p = 0.05 significance level (two-tailed).

## Results

### Corneal Anterior Radii

**Figure 3:**
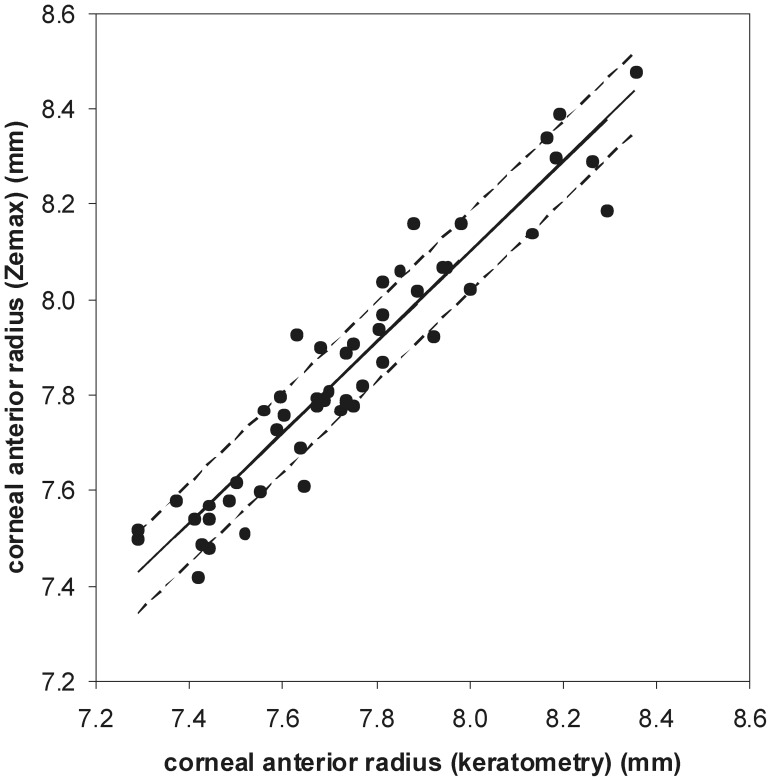
Linear fit between anterior corneal radii calculated by Zemax® and evaluated by keratometry. Regression parameters y (σ = 0.084) = 0.948 (σ = 0.045)x+0.516 (σ = 0.347). Pearson correlation parameters: r = 0.949, p<0.001.

To test the efficacy of the representation of the cornea in our model, corneal anterior radii of the Zemax® representation of the 104 corneas were evaluated before refractive surgery and compared to values obtained by keratometry. In Figure 3 it is shown that there was a very strong correlation between the corneal anterior radii calculated by Zemax® and evaluated by keratometry. There was a difference in mean values, with anterior radii determined by Zemax® having a higher mean value than those determined by keratometry – Table 1.

**Table 1:** Analysis of parameters calculated by our model and chosen comparators.

	mean±sd	Mean absolute difference	Median	Median absolute difference	
**Corneal anterior radii (mm)**					
keratometry	7.74±0.26*	0.12	7.72	0.12	
Our model	7.85±0.26*		7.80		
**ACDpost (mm)**					
Olsen 2	4.87±0.24*	0.36	4.86	0.34	
Our model	5.22±0.23*		5.22		
**Pre-op IOL (D)**					
SRK/T	17.2±2.9	0.6	17.4	0.5	
Our model	16.9±2.8		17.5		
**Post-op IOL (D)**					
Average IOL	21.8±1.6	0.9	22.0	0.5	
Our mode	21.5±1.7		21.5		

* p<0.05 compared to our model, unpaired t-test.

### Anterior Chamber Depth Estimation

**Figure 4:**
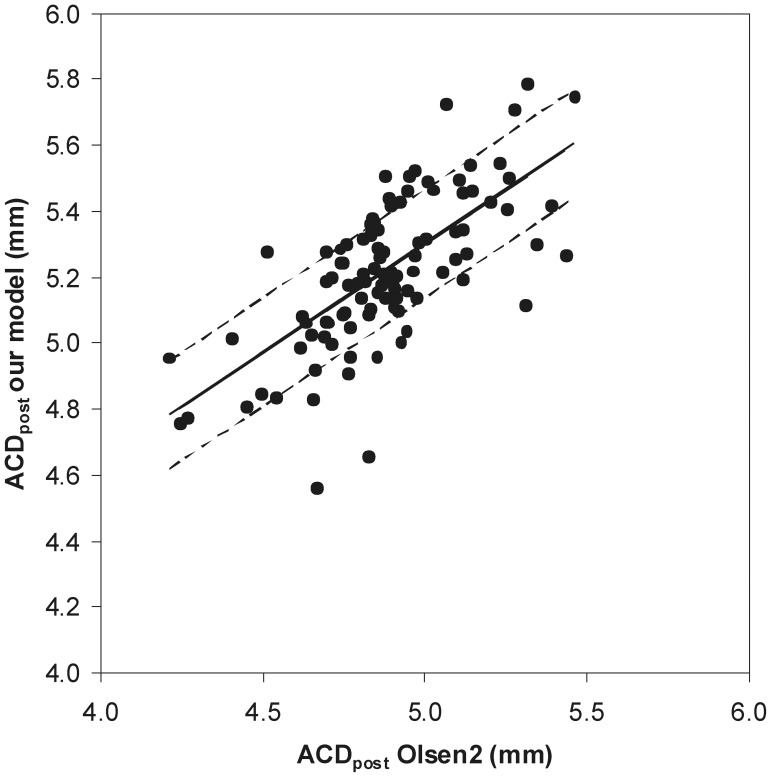
Linear fit between ACD_post_ estimation using our model and the Olsen 2 formula. Regression parameters y (σ = 0.164) = 0.657 (σ = 0.069)x+2.014 (σ = 0.335). Pearson correlation parameters: r = 0.688, p<0.001.

The value of ACD_post_ is necessarily an estimation, needed for post-operative IOL power calculation. ACD_post_ calculation using our model used solely lens and anterior chamber biometric values, as previously described in the Methods Section. In order to validate the calculated values, we have tested the correlation between our ACD_post_ estimation and values obtained using the Olsen 2 formula, transformed to ACD prediction algorithm as described by Jin et al [7], which also uses corneal thickness and anterior chamber depth data. A strong correlation was obtained (Figure 4). Mean values were statistically different, with ACD_post_ (Olsen2) having a mean value lower than ACD_post_ (our model) – Table 1.

### Pre-operative IOL Power

**Figure 5:**
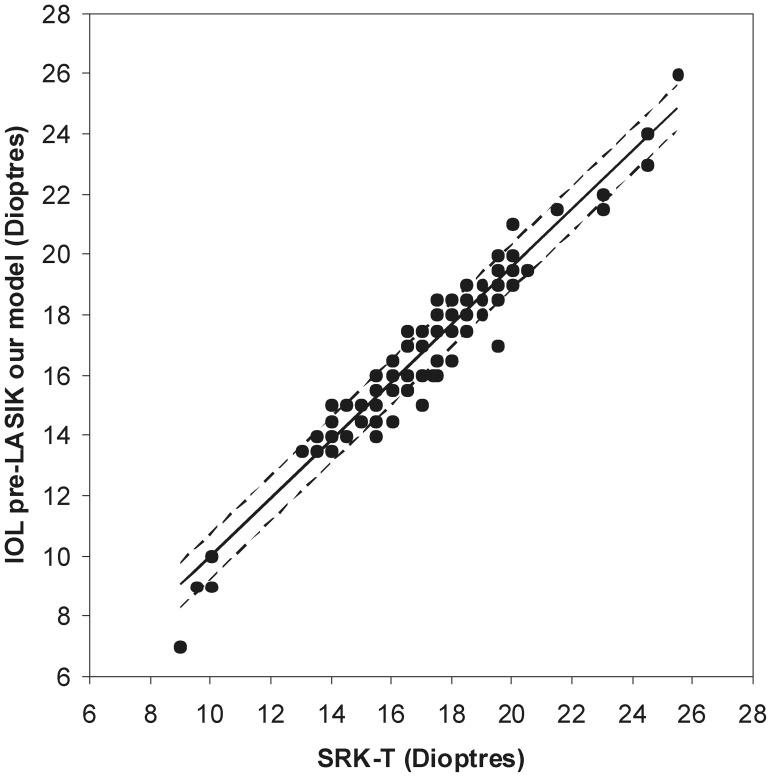
Linear fit between pre-operative IOL power estimation using our model and using the SRK-T formula. All values were rounded to 0.5 dioptres, in order to reflect currently available IOL powers. Regression parameters y (σ = 0.745) = 0.959 (σ = 0.026)x+0.409 (σ = 0.446). Pearson correlation parameters: r = 0.966, p<0.001.

In order to validate the pre-operative IOL power estimated using our model, we have analysed the correlation between the values obtained and the ones calculated using the Sanders-Retzlaff-Kraft-Theoretical (SRK-T) formula, as well as the differences between the mean values. The SRK-T formula was used as a comparator since it is the most frequently used for IOL power calculations. There was a very strong correlation between pre-operative IOL power estimation using our model and using the SRK-T formula – Figure 5– with no difference of mean values – Table 1.

### Post-operative IOL Power

**Figure 6:**
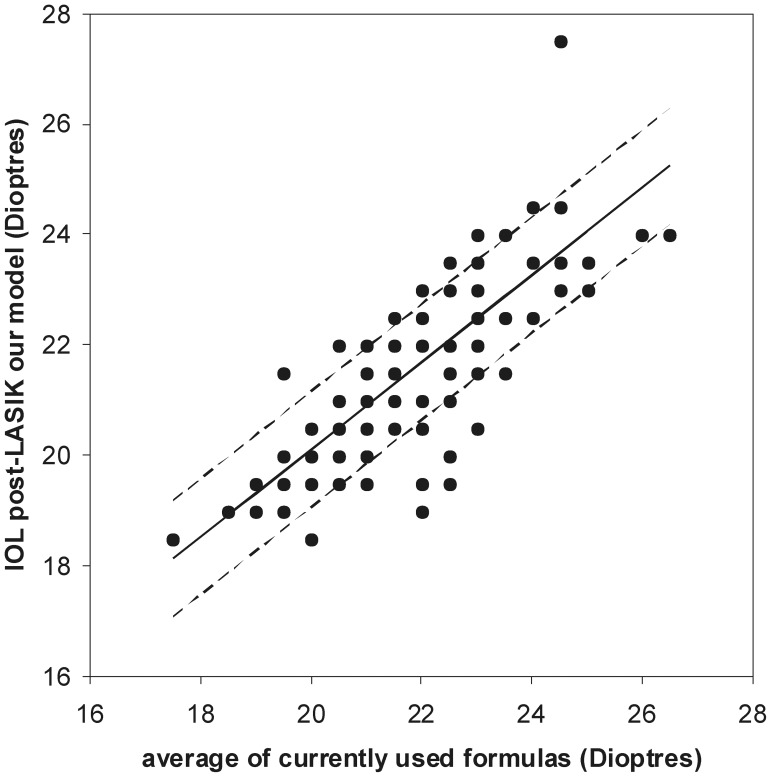
Linear fit between post-LASIK IOL using our model and the average of currently used formulas. All values were rounded to 0.5 dioptres, in order to reflect currently available IOL powers. Regression parameters y (σ = 1.048) = 0.788 (σ = 0.063)x+4.340 (σ = 1.381). Pearson correlation parameters: r = 0.778, p<0.001.

The SRK-T formula has been shown not to be accurate for post- LASIK IOL power calculation, and of the several currently used methods, the ones using surgically induced changes in manifest refraction or using no prior data have been shown to have smaller IOL prediction errors and variances and greater percentages of eyes within ±0.50 and ±1.00 D of the refractive prediction errors [3]. Since, among these methods, none has proven to be better [3], we have chosen as a comparator for post-operative IOL power the method that uses the mean IOL power, called average IOL power in the calculator available at the ASCRS website [29]. Results have shown a strong correlation (Figure 6), with no difference of mean values – Table 1.

## Discussion

Although wavefront technology and exact ray-tracing have been used for IOL power calculations [5]–[7], the model proposer in this paper has some differences, in that we have incorporated modifications, aiming at improving the representation of the pseudophakic eye: 1) a full definition of corneal elevation data, obtained from topography, was used, as opposed to only corneal radii, so that the model is prepared to represent irregular corneas, 2) ACD_post_ estimation was based solely on biometric values and 3) in order to evaluate the best image plane, a metric based on the MTF and CSF was chosen, thus allowing the use of real ray-tracing, instead of the more common paraxial ray-tracing, and a grid of rays, instead of only one ray.

Comparison of corneal anterior radii values calculated by Zemax® and evaluated by keratometry not only showed a very strong correlation but also no statistically significant difference in mean values. The difference of 0.5% was below 1.5%, which is the percentage described by Preussner [5] when considering asphericity. Keratometric assessment measures only the corneal central area, relying on the assumption that, being a spherical surface, the measurement will be the same regardless of the area from which it is taken. This supposition is not entirely true since even physiological corneas have asphericities, and being particularly relevant in the case of a pseudophakic eye, which presents considerable positive spherical aberration. The corneal representation we have chosen to use on our eye model incorporates not only anterior and posterior radii, with their asphericity and toricity, but also their irregularities, thus aiming at an enhancement of the accuracy of anterior radii calculation. Analysis of the best curve fit parameters show that the error associated with regression values estimation is very small, varying between 1.0% and 1.1%. Of notice is the fact that being A = 0.516±0.347 and B = 0.948±0.045, these values become very close to 0 and 1, respectively, thus strengthening the validity of our eye model. We have chosen an Orbscan II® for topography data due to being the most widely used in clinical practice, but a possible future improvement of this model could be achieved by using interferometry data, which are more accurate than ultrasonography [30]. Considering the mathematical and population free nature of our model, it is prepared to immediately incorporate new data as they become available, such as more accurate measurements of any eye element, with the consequent immediate improvement of outcome.

Another extremely important parameter is ACD_post_ estimation. Its importance on refractive result is well established, since ACD_post_ prediction error accounts for 42% of all sources of error on IOL power calculation [8], thus being one of the main sources of error. ACD_post_ will always be an estimation, since it cannot be physically measured before IOL placement, and moreover it varies depending on the surgical technique used and behavior of the complex capsular bag-IOL. However, it was our purpose to test a simple model that would estimate ACD_post_ independently of population averages, based solely on biometric values and on the known geometry of the implanted IOL. In order to test our method of ACD_post_ estimation, we have correlated it with the Olsen 2 formula, which is the most widely used for this calculation.

Our eye model showed a strong correlation with ACD_post_ estimation using the Olsen 2 formula, with error associated with regression values estimation varying between 3.0% and 3.9%, when considering the ACD_post_ values of our population sample, corresponding to an error of 0.25 dioptres in refractive error [19]. However, the ACD_post_ estimated by our eye model showed a 6.6% statistically significant difference in mean values in comparison with the Olsen 2 formula.

These results may be explained due to the fact that the Olsen 2 empirical formula, which also correlates with lens thickness and ACD, uses the Gaussian approximation of the “effective lens position” and not the physical position of the IOL.

Our eye model estimates ACD_post_ based solely on biometric values – ACD_pre_ and lens thickness –, enhancing the equator definition by using the derivative of population studies which are the base for the Liou-Brennan model [13]. Also, in our eye model, IOL is described by its geometry – anterior and posterior radii, asphericity of the surfaces, thickness and refraction index – thus taking into account the specificity of the type of IOL used, which influences its intraocular position. The definition of the physical intraocular position of the IOL, as included in our model, raises the possibility of improving its estimation by allowing its comparison with post-surgery biometric measurements, as these become more accurate.

The comparison of pre-LASIK IOL power using our eye model with the SRK-T formula showed a very strong correlation, with no difference in mean values. The error associated with regression values estimation varied between 2.9% and 8.3%, when considering the pre-LASIK IOL values of our population sample, representing a standard deviation of 0.53 D. Considering the available IOLs vary in steps of 0.5 D, which we have taken into account on our linear fitting, and IOL manufacturers should apply an internal tolerance of ±0.25 D to all IOLs [19], then the error of 0.53 D from our model falls well within this range. This very strong correlation with no difference in mean values strengthens the validity of our model, in suggesting that our eye model is comparable to the SRK-T formula, which has been shown to be appropriate for IOL power calculation in eyes that fall within population averages, which comprise 75% of all cases [31]. Also, being A = 0.409±0.446 and B = 0.959±0.026, these values become very close to 0 and 1, respectively, and further support the validity of our eye model. However, IOL power estimation of the SRK-T formula in eyes previously subject to refractive surgery is poor and inadequate [32], and although several formulas have been proposed to improve IOL power calculation in “abnormal” eyes, either by using pre-operative clinical data [33]; [34], correction factors [35]; [36] or normograms [37], none has proven to be more effective or accurate than another.

Given the above, we have chosen as a comparator the average IOL power in the calculator available at the ASCRS website [29], since this is the recommendation for clinical practice [3]. Results showed a strong correlation, with no difference in mean values. The error associated with regression values estimation varied between 4.0% and 6.0%, when considering the post-LASIK IOL values of our population sample, representing a standard deviation of 0.62 D, and again falling well within the range previously calculated based on Norrby [19]. A less strong correlation in the case of post-LASIK IOL power calculation was expected, since the comparison was done with the average IOL power, which shows important inter-formula values differences.

The reality that a perfect solution for post-LASIK IOL power estimation is yet to be attained underscores the importance of seeking new methods to determine IOL in pseudophakic eyes. Currently used formulas are only applicable to eyes that fall within population average values and if using the technologies they were developed for, and thus, whenever one of these variables change, new population studies are needed, leading to a time-gap that leaves clinical practice without immediate solutions.

Results show that our eye model is applicable to IOL power calculation, both pre- and post-LASIK, in a personalized way, without the need for population averages, which in cases such as post-LASIK may be very different from the general population.

The modelling of a human pseudophakic eye not only has multiple current clinical applications but may also be used for future diagnostic and correction challenges. Several relevant factors are still unknown, but the results presented in this paper suggest that the development of these eye models, considering individual aberrations, using wavefront technology and exact ray-tracing, enhanced by the image metric based on MTF and CSF, allowing for the prompt incorporation of parameters that are currently not measurable in clinical practice, in a personalized manner, if and when they become available, without the need for re-defining population correction factors, can be used even in face of abnormal corneas or when clinical history is unknown.
